# Supplementary figures and images for: The Effect of Waning on Antibody Levels and Memory B Cell Recall following SARS-CoV-2 Infection or Vaccination
Source: Vaccines (Basel). 2022 Apr 29;10(5):696. doi: 10.3390/vaccines10050696 (PMC9143792; doi:10.3390/vaccines10050696)

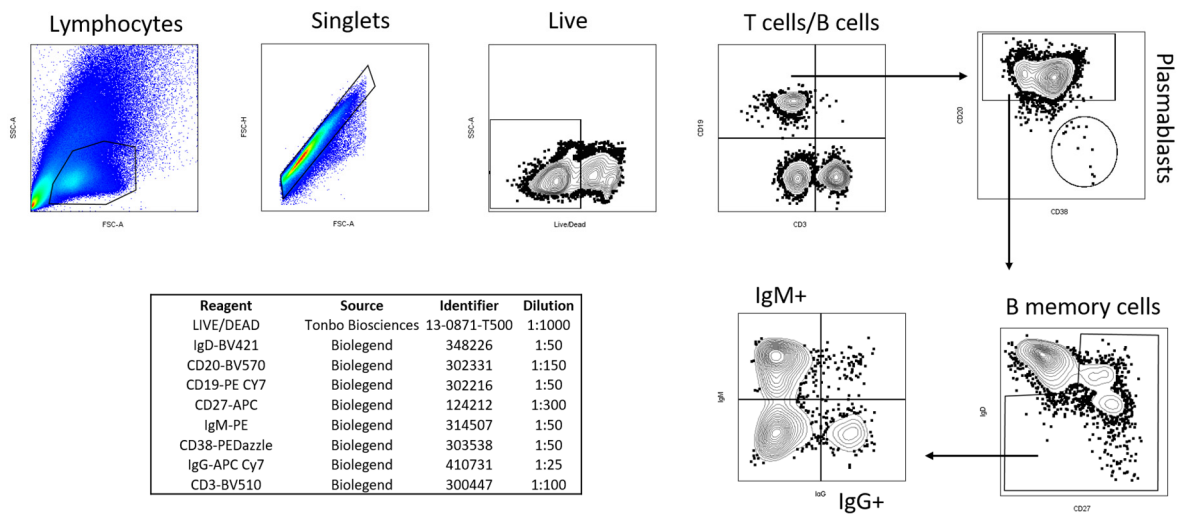

**Figure S1.** Memory B cell gating strategy.

Supplement: Supplementary file 1 [file vaccines-10-00696-s001.zip › Figure S1.pdf]
